# Comparative Analysis of Volatile Components in Chi-Nan and Ordinary Agarwood Aromatherapies: Implications for Sleep Improvement

**DOI:** 10.3390/ph17091196

**Published:** 2024-09-11

**Authors:** Zixiao Jiang, Junyu Mou, Jian Feng, Shunan Zhang, Dan Li, Yangyang Liu

**Affiliations:** 1 Hainan Provincial Key Laboratory of Resources Conservation and Development of Southern Medicine, Key Laboratory of State Administration of Traditional Chinese Medicine for Agarwood Sustainable Utilization; International Joint Research Center for Quality of Traditional Chinese Medicine; Hainan Branch of Institute of Medicinal Plant Development, Chinese Academy of Medical Sciences; Peking Union Medical College, Haikou 570311, China; jiangzixiao98@163.com (Z.J.); mjy5197664@163.com (J.M.); jianfenghn@126.com (J.F.); s2023009013@student.pumc.edu.cn (S.Z.); 2 The Burdon Sanderson Cardiac Science Centre and BHF Centre of Research Excellence, Department of Physiology, Anatomy and Genetics, University of Oxford, Oxford OX1 3PT, UK; dan.li@dpag.ox.ac.uk

**Keywords:** agarwood, aromatherapy, network pharmacology, sleep aid, molecular docking, bioinformatics

## Abstract

Agarwood, a precious traditional medicinal herb and fragrant material, is known for its sedative and sleep-improving properties. This study explores the mechanisms underlying the aromatherapy effects of Chi-Nan agarwood and ordinary agarwood in improving sleep. Using a combination of gas chromatography–mass spectrometry (GC-MS), network pharmacology, and molecular docking techniques, we identified and c ompared the chemical compositions and potential molecular targets of both types of agarwood. The GC-MS analysis detected 87 volatile components across six types of agarwood aromatherapy, with 51 shared between Chi-Nan and ordinary agarwood, while each type also had 18 unique components. Ordinary agarwood was found to be richer in sesquiterpenes and small aromatic molecules, whereas Chi-Nan agarwood contained higher levels of chromones. These differences in chemical composition are likely responsible for the distinct sleep-improving effects observed between the two types of agarwood. Through network pharmacology, 100, 65, and 47 non-repetitive target genes related to sleep improvement were identified for components shared by both types of agarwood (CSBTs), components unique to common agarwood (CUCMs), and components unique to Chi-Nan agarwood (CUCNs), respectively. The constructed protein–protein interaction (PPI) networks revealed that key targets such as *MAOA*, *MAOB*, *SLC6A4*, and *ESR1* are involved in the sleep-improving mechanisms of agarwood aromatherapy. Molecular docking further confirmed the strong binding affinities of major active components, such as 5-Isopropylidene-6-methyldeca-369-trien-2-one and 2-(2-Phenylethyl)chromone, with these core targets. The results suggest that agarwood aromatherapy enhances sleep quality through both hormonal and neurotransmitter pathways, with ordinary agarwood more deeply mediating hormonal regulation, while Chi-Nan agarwood predominantly influences neurotransmitter pathways, particularly those involving serotonin and GABA. This study provides valuable insights into the distinct therapeutic potentials of Chi-Nan and ordinary agarwood, highlighting their roles in sleep improvement and offering a foundation for future research in the clinical application of agarwood-based aromatherapy.

## 1. Introduction

Insomnia, characterized by difficulty in falling or staying asleep, has become a prevalent clinical issue in modern society. It is often triggered by various factors, including emotional stress and occupational demands, leading to a significant number of individuals suffering from poor sleep quality [1]. The traditional clinical approach to managing insomnia involves the use of sedative–hypnotic drugs. However, these medications are often accompanied by adverse side effects, including drug dependence, tolerance, withdrawal symptoms, and even potential rebound insomnia upon discontinuation [2,3]. As a result, there is a growing interest in finding safe and effective alternatives to pharmacological treatments for sleep disorders.

Agarwood, known for its unique and valuable fragrance, has been utilized for centuries in traditional medicine and aromatherapy across various cultures. Often referred to as the “king of incense,” agarwood is highly prized in regions such as China, the Middle East, Europe, and the United States for its therapeutic properties [4]. Historically, agarwood was used [5] in China as early as the Sui and Tang dynasties, where it was burned in court settings to signify solemnity and respect [6]. Today, with advancements in cultivation and processing technologies, agarwood is broadly categorized into two main types: ordinary agarwood and Chi-Nan agarwood [7]. These two varieties of agarwood exhibit distinct aromatic profiles and are believed to offer different therapeutic benefits, particularly in terms of calming the mind and improving sleep [8].

Previous studies have suggested that agarwood aromatherapy has a sedative effect, potentially aiding in sleep improvement by influencing neurotransmitter levels and neural activity. In our preliminary animal experiments, we found that the inhalation of agarwood incense significantly inhibited the spontaneous activity of insomnia-induced mice, effectively improving their sleep conditions. The mechanism of action may be related to the regulation of 5-HT neurotransmitter levels in the brain, balancing GABA-Glu secretion, and the synthesis, metabolism, and transport of Glu [3,9,10]. Despite these insights, there remains a lack of comprehensive understanding regarding the specific mechanisms through which Chi-Nan agarwood exerts its sleep-promoting effects.

To address this gap in knowledge, the current study aims to explore the similarities and differences between Chi-Nan agarwood and ordinary agarwood in improving sleep quality. By employing a combination of gas chromatography–mass spectrometry (GC-MS) for chemical analysis and network pharmacology, the study seeks to identify the active components of these agarwood varieties and elucidate their mechanisms of action. This research will provide important references for the clinical application of agarwood aromatherapy and its potential optimization in the broader health industry.

## 2. Results and Discussions

### 2.1. Composition Analysis of Agarwood Aromatherapy

NIST SEARCHER 2.4 was used in conjunction with the NIST17 database to search for the constituents of each agarwood aromatherapy obtained by GC-MS, and only compounds with a match degree of 60 or higher were retained. Eighty-seven volatile constituents were detected in the six types of agarwood aromatherapy. The two types of agarwood have a total of 51 components, and there are 18 components unique to ordinary agarwood and 18 components unique to Chi-Nan agarwood (Table 1).

**Figure 1:**
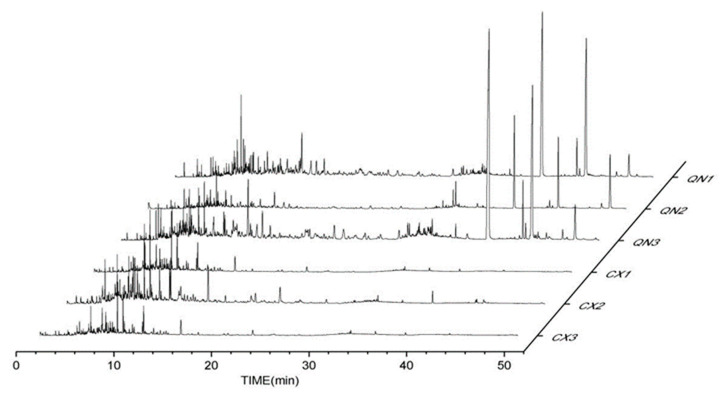
GC-MS total ion flow diagrams of different agarwood aromatherapies. (CX1: common agarwood aromatherapy; CX2: common agarwood aromatherapy; CX3: common agarwood aromatherapy; QN1: Chi-Nan agarwood aromatherapy; QN2: Chi-Nan agarwood aromatherapy; QN3: Chi-Nan agarwood aromatherapy).

Agarwood aromatherapies are mainly composed of small aromatic substances, sesquiterpene components, chromones, and fatty acid components. The peak times of the volatile components in the common agarwood aromatherapy (Figure 1) were mainly concentrated in 4~18 min, while the peak times of the Chi-Nan agarwood aromatherapy were more uniform, and two chromones with larger peaks appeared in 40~50 min. In addition to the CSBTs, the CUCMs include five sesquiterpenes, four small molecules of aromatic substances, one fatty acid, and eight kinds of chromones. The CUCNs include six kinds of chromones, one small molecule of aromatic substances, and eleven kinds of sesquiterpenes.

**Figure 2:**
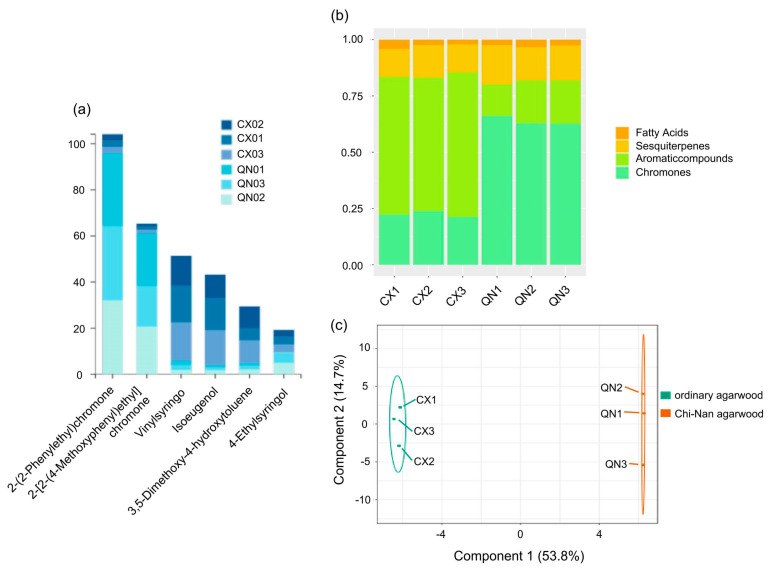
Comparison of compounds and relative content of ordinary agarwood aromatherapy and Chi-Nan agarwood aromatherapy ((**a**) Percentage of the top 6 compounds in each group of agarwood aromatherapy; (**b**) types of compounds and their percentage in each group of agarwood aromatherapy; (**c**) S-PLOT plots of different types of agarwood aromatherapy).

In order to outline the relationships and differences between groups, we used OPLS-DA plots to describe them. In the OPLS-DA (Figure 2c), the t [1] accounted for 53.8% of the sample variance and t [2] accounted for 14.7% of the sample variance. The results indicated that the components of the three types of agarwood aromatherapy of the same type were basically similar, while the compositions of different types of agarwood aromatherapy vary greatly. The relative content of small-molecule aromatic substances in CUCMs is about 60%, with fewer chromone components (about 20%). In contrast, the proportion of aromatic substances decreases sharply (about 20%) and the chromone content increases sharply (about 60%) in the CUCNs. At the same time, the characteristic sesquiterpenoids are widely present in both types of agarwood aromatherapy and are present in roughly equal amounts; in addition, there are some small amounts of fatty acids (Figure 2b). Among the chemical constituents of different agarwood aromatherapies, the top six constituents are 2-(2-Phenylethyl)chromone, 2-[2-(4-Methoxyphenyl)ethyl]chromone, Vinylsyringo, Isoeugenol, 3,5-Dimethoxy-4-hydroxytoluene, and 4-Ethylsyringol, of which the two 2-(2-Phenylethyl)chromones unique to agarwood occupy the top two places. These two 2-(2phenylethyl)chromone components are present in both ordinary agarwood aromatherapy and Chi-Nan agarwood aromatherapy, but the content in the former is much lower than that in the latter (Figure 2a). For 2-(2-Phenylethyl)chromone, it was contained in the amounts of 2.54%, 2.86%, and 2.82% in the three kinds of common agarwood aromatherapy, while it reached 31.65%, 32.04%, and 32.20% in the three kinds of Chi-Nan agarwood aromatherapy; 2-[2-(4-Methoxyphenyl)ethyl] chromone only occupied 1.20%, 1.34%, and 1.58% in the three kinds of common agarwood aromatherapy, while it reached 22.83%, 20.56%, and 17.78% in the three kinds of Chi-Nan agarwood aromatherapy.

### 2.2. Confirmation of Active Ingredients in Agarwood Aromatherapy and Construction of Target Genealogy Set

According to the GC-MS results screened in the TCMSP and PubChem databases, there are a total of 48 effective standardized active substances for the CSBTs, 16 active ingredients for the CUCMs, and 15 active ingredients for the CUCNs (Table 1). By integrating the target data, 589, 323, and 220 potential targets were obtained in the three parts, respectively. Using ‘Sleep disorders’ as a search term (this keyword also contains all the targets of insomnia), 636 sleep disorder-related genes were obtained by integrating multiple databases according to the screening criteria.

### 2.3. Protein-Protein Interaction Network Construction and Analysis

**Figure 3:**
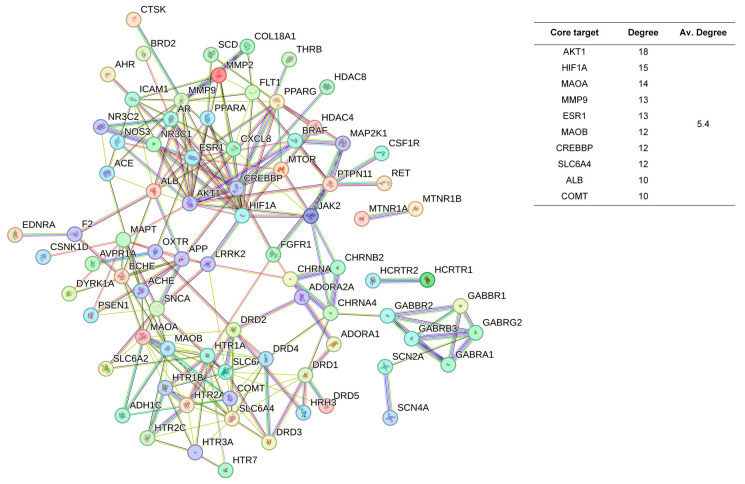
PPI network diagram and core target information for the CSBTs.

**Figure 4:**
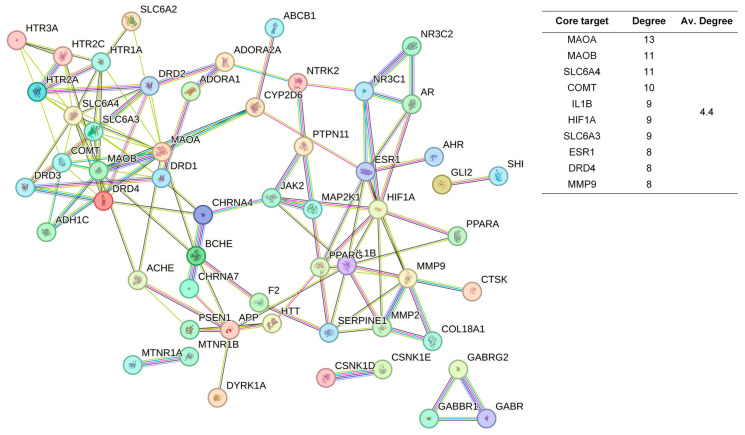
PPI network diagram and core target information for the CUCMs.

**Figure 5:**
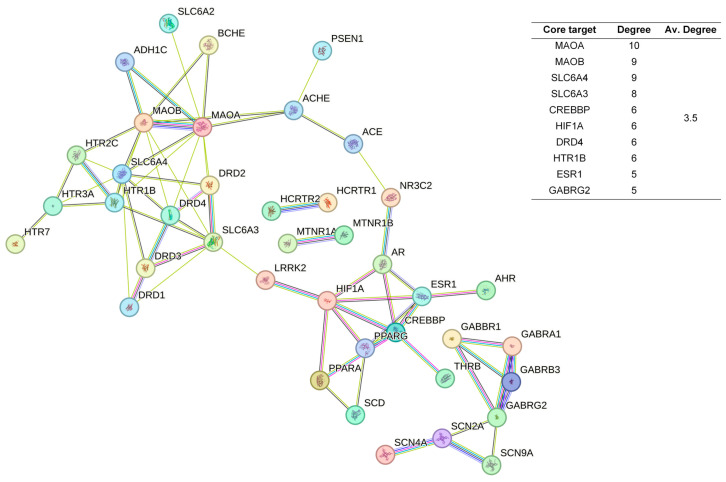
PPI Network diagram and core target information for the CUCNs.

The 48 standardized components of the CSBTs (Figure 3) have 100 non-repetitive intersecting sets of target genes with the spectrum of targets related to improved sleep, based on the STRING protein interactions background network to construct a PPI network of 100 potential target genes for sleep improvement. The network contained 100 nodes and 220 edges with an average node degree value of 4.4. According to the enrichment analysis, the PPI enrichment *p*-value < 1.0 × 10^−16^ was shown to be able to be significantly enriched. Therefore, the selected 100 proteins can be made as a whole and partially connected biologically. Similarly, the 16 active ingredients for the CUCMs and the target genealogical set of improving sleep had 65 intersecting targets, and the PPI network contained 65 nodes and 121 edges, with an average node degree value of 3.72 (Figure 4). The 15 active ingredients for the CUCNs and the target genealogical set of CUCMs had 47 intersecting genes, with 47 nodes and 79 edges, with an average node degree value of 2.98 (Figure 5).

Among the top 10 core targets identified in the three sections of the study, six key genes were specifically associated with ordinary agarwood: *MAOA*, *MAOB*, *SLC6A3*, *SLC6A4*, *HIF1A*, and *ESR1*. These genes play critical roles in various biological processes essential for the regulation of neurotransmission, cellular metabolism, and stress responses. *MAOA* (Monoamine oxidase A) and *MAOB* (Monoamine oxidase B) are enzymes that metabolize neuroactive and vasoactive amines within the central nervous system (CNS) and peripheral tissues. *MAOA* primarily catalyzes the oxidative deamination of primary amines, including vital neurotransmitters such as serotonin (5-HT), dopamine (DA), and norepinephrine (NE), with a preference for oxidizing 5-HT [11,12,13]. This process is crucial for the degradation and regulation of neurotransmitter levels, thereby influencing mood, behavior, and overall neural function. MAOB, while functionally similar, preferentially degrades benzylamine and phenylethylamine, playing a significant role in maintaining neurotransmitter balance and modulating physiological processes like mood regulation, and motor control [14]. *SLC6A3*, also known as the dopamine transporter (DAT), and *SLC6A4*, the serotonin transporter (SERT), are both critical transport proteins. *SLC6A3* is responsible for the reuptake of dopamine into presynaptic neurons, effectively terminating its action and regulating dopaminergic signaling, which is integral to reward mechanisms, motor function, and cognitive processes [15]. *SLC6A4*, on the other hand, manages the reuptake of serotonin from the synaptic cleft back into the presynaptic neurons, ensuring proper serotonergic signaling, which is vital for mood stabilization, anxiety control, and sleep regulation [16,17,18]. *HIF1A* (Hypoxia-inducible factor 1-alpha) serves as a major transcriptional regulator in response to hypoxic conditions, activating genes that enable cellular adaptation to low oxygen levels, influencing processes like angiogenesis, metabolism, and cell survival [19]. *ESR1* (Estrogen receptor 1) and its receptor are pivotal in regulating gene expression influenced by estrogen, affecting cell proliferation, differentiation, and survival, particularly in reproductive tissues, while also playing roles in bone health, cardiovascular function, and brain activity [20]. Collectively, these genes underscore the multifaceted mechanisms through which ordinary agarwood may exert its therapeutic effects, particularly in modulating neurotransmitter activity and hormonal responses, thereby contributing to improved mental health and overall well-being.

In addition to the six shared gene targets identified, ordinary agarwood aromatherapy uniquely targets core genes such as *COMT* (catechol-O-methyltransferase) and IL1B, which play significant roles in sleep regulation. *COMT* is an enzyme that catalyzes the O-methylation of catecholamines, including neurotransmitters like norepinephrine, epinephrine, and dopamine, as well as catechol hormones. This process inactivates these molecules, effectively regulating their levels and shortening the biological half-life of certain neuroactive drugs, such as levodopa, α-methyldopa, and isoproterenol. *COMT* belongs to the class I SAM-binding methyltransferase superfamily and is crucial for maintaining neurotransmitter balance, particularly in relation to stress and sleep disorders. IL1B, another key target, is involved in the inflammatory response and has been linked to the modulation of sleep through its effects on immune function and cytokine production [21,22,23].

In contrast, Chi-Nan agarwood aromatherapy uniquely targets sleep-aiding genes such as *HTR1B* (5-hydroxytryptamine receptor 1B) and *GABRG2* (γ-aminobutyric acid receptor subunit γ-2). *HTR1B* is a G-protein-coupled receptor for serotonin (5-HT) that plays a vital role in regulating mood, anxiety, and sleep by modulating serotonergic signaling pathways. *GABRG2* is a subunit of the GABA-A receptor, which is a ligand-gated chloride channel. This subunit is essential for the formation of functional inhibitory GABAergic synapses and mediates synaptic inhibition as a GABA-gated ion channel. The activation of GABA-A receptors by *GABRG2* is critical for inducing relaxation and sleep by reducing neuronal excitability [24,25]. Together, these unique targets highlight the distinct molecular pathways through which different types of agarwood aromatherapy exert their sleep-enhancing effects, with ordinary agarwood focusing on neurotransmitter metabolism and Chi-Nan agarwood on neurotransmitter receptor modulation.

Therefore, based on the analysis of PPI network construction, it can be tentatively concluded that agarwood aromatherapy mainly regulates hormones and neurotransmitters to improve sleep quality.

### 2.4. GO and KEGG Analysis

**Figure 6:**
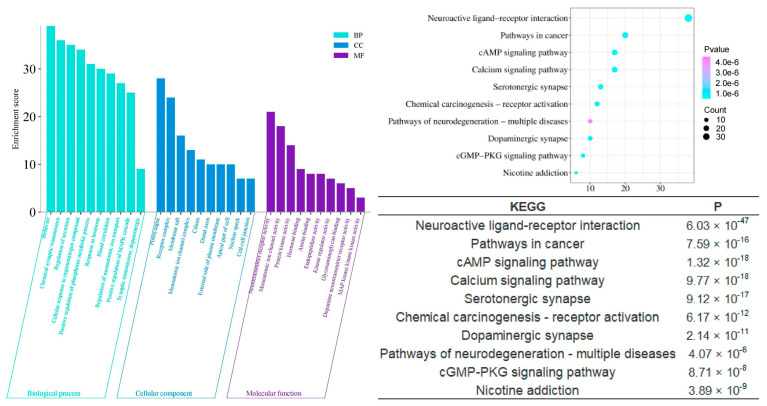
GO and KEGG analysis of CSBTs.

To scientifically and systematically explore the mechanism by which agarwood aroma improves sleep, we conducted a Gene Ontology (GO) Function Enrichment and KEGG Pathway Enrichment analysis to identify potential targets involved in sleep enhancement. The analysis revealed that the shared components of agarwood are primarily involved in several key biological processes (Figure 6), including chemical synaptic transmission (*p* = 6.03 × 10^−38^), regulation of secretion (*p* = 1.07 × 10^−38^), positive regulation of the phosphorus metabolic process (*p* = 9.55 × 10^−31^), and response to hormones (*p* = 5.25 × 10^−15^). In terms of cellular components, these shared components are significantly associated with postsynaptic structures (*p* = 1.59 × 10^−19^), receptor complexes (*p* = 1.15 × 10^−15^), and monoatomic ion channel complexes (*p* = 2.14 × 10^−6^). On the molecular function level, the components play roles in monoatomic ion channel activity (*p* = 2.40 × 10^−26^), neurotransmitter receptor activity (*p* = 5.75 × 10^−24^), protein kinase binding (*p* = 7.94 × 10^−6^), hormone binding (*p* = 1.17 × 10^−5^), and dopamine neurotransmitter receptor activity (*p* = 1.17 × 10^−5^). These findings suggest that agarwood aroma may improve sleep through modulating neurotransmission, receptor activity, and hormonal responses at multiple levels of cellular function.

**Figure 7:**
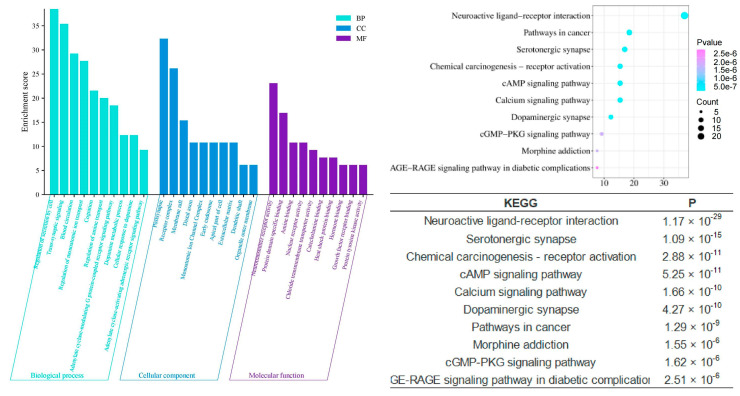
GO and KEGG analysis of CUCMs.

Compared to the multifaceted and multidimensional effects of the shared components, the enrichment analyses of the unique components of different agarwood aromatherapy products provide deeper insights into their specific intra-human activities related to sleep improvement. The unique components of common agarwood (CUCMs) appear to have a stronger involvement in hormonal regulation, as evidenced by significant enrichment in gene ontology functions such as response to hormones (*p* = 1.23 × 10^−26^) and regulation of hormone levels (*p* = 1.32 × 10^−12^). These functions suggest that CUCMs may influence the endocrine system, thereby playing a role in modulating sleep through hormonal pathways. On a molecular functional level, CUCMs are significantly associated with activities such as nuclear receptor activity (*p* = 1.86 × 10^−14^), catecholamine binding (*p* = 4.90 × 10^−11^), adrenergic receptor activity (*p* = 7.76 × 10^−7^), hormone binding (*p* = 3.39 × 10^−5^), and protein kinase activator activity (*p* = 2.45 × 10^−3^). These activities highlight the potential for CUCMs to interact with key hormonal receptors and signaling molecules, thereby influencing various hormonal processes that could be linked to sleep regulation (Figure 7).

**Figure 8:**
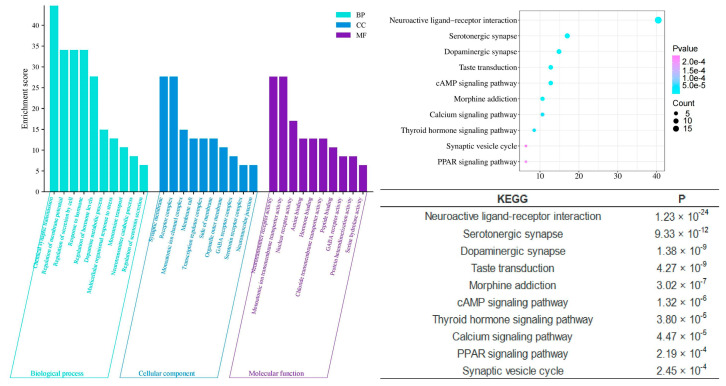
GO and KEGG analysis of CUCNs.

In contrast, the unique components of Chi-Nan agarwood (CUCNs) are more closely associated with neurotransmitter-related interventions, which are crucial for the modulation of sleep. Enrichment analyses reveal significant involvement in processes such as chemical synaptic transmission (*p* = 3.09 × 10^−27^), dopamine metabolic processes (*p* = 2.69 × 10^−14^), regulation of neurotransmitter levels (*p* = 5.01 × 10^−15^), neurotransmitter catabolic processes (*p* = 1.05 × 10^−9^), and regulation of serotonin secretion (*p* = 1.23 × 10^−7^). These processes suggest that CUCNs may enhance sleep by modulating neurotransmitter systems, particularly those involving dopamine and serotonin, which are well-known regulators of mood and sleep cycles. Furthermore, cellular components enriched in CUCNs include the GABA receptor complex (*p* = 2.95 × 10^−8^), serotonin receptor complex (*p* = 2.95 × 10^−7^), and neurotransmitter receptor activity (*p* = 2.75 × 10^−22^). These findings indicate that CUCNs may have a direct impact on neurotransmitter receptor function, potentially enhancing inhibitory neurotransmission through GABA receptors or modulating excitatory neurotransmission via serotonin receptors. Molecular functions such as amine binding (*p* = 9.12 × 10^−15^) and GABA receptor activity (*p* = 3.63 × 10^−8^) further support this neurotransmitter-centric activity, suggesting that CUCNs could provide a calming effect conducive to sleep (Figure 8).

The KEGG pathway enrichment analysis further elucidates the mechanisms by which agarwood aromatherapy improves sleep, focusing on therapeutic pathways. Among the top 10 significantly enriched KEGG pathways across the three-part component analysis, five pathways are consistently present: neuroactive ligand-receptor interaction, the cAMP signaling pathway, serotonergic synapses, dopaminergic synapses, and morphine addiction. These pathways highlight the major metabolic processes involved in the regulation of sleep disorders through agarwood aromatherapy. Dopaminergic synapses, a type of chemical synapse, play a critical role in affective disorders and can influence the differential connectivity of transmembrane molecules belonging to the axonemal protein superfamily, which are essential in neuropsychiatric disorders and excitatory cell function [26]. Serotonergic synapses, similar to dopaminergic synapses, are chemical synapses but with a broader distribution throughout the body, particularly within the cerebral cortex [27]. These synapses are key players in regulating mood and sleep. Neuroactive ligand-receptor interactions can modulate dopamine (DA) levels, thereby affecting mood and memory functions, which are crucial for sleep regulation [28]. The cAMP signaling pathway, also known as the PKA system, involves cyclic nucleotides where extracellular signals bind to corresponding receptors, activating PKA to phosphorylate downstream target proteins by regulating intracellular cAMP levels [29]. This pathway influences cellular metabolism and behavior, which can have direct implications for sleep patterns.

Compared to the signaling pathways involved in the unique components of common agarwood (CUCMs), the unique components of Chi-Nan agarwood (CUCNs) are more involved in neurotransmitter-based pathways, such as the calcium signaling pathway, PPAR signaling pathway, and synaptic vesicle cycle. The calcium signaling pathway is closely related to insomnia, with Ca^2+^ playing a role in regulating various neuronal functions, such as neurotransmitter release, cell structure, and enzyme system activation [30,31]. When the body experiences symptoms of insomnia, the molecules associated with excitatory signaling pathways become elevated, enhancing the conduction of these pathways. The synaptic vesicle cycle refers to the process by which neurotransmitters stored in synaptic vesicles are released through regulated exocytosis to mediate synaptic transmission [12,32,33]. This cycle is crucial for maintaining effective communication between neurons, which is essential for normal sleep function.

Therefore, with the analysis of the GO and KEGG enrichment results of the different agarwood aromatherapies, the mechanism of action of different agarwood aromatherapies to improve sleep is becoming clearer and clearer. On one hand, based on the above results, it is surmised that the active ingredients of agarwood aromatherapy take *ESR1*, *AKT1*, and other protein receptors as the core targets by affecting the metabolism and proliferation of hormone cells and interfering with the kinase-binding channel and affect the proliferation and differentiation of cells in the target tissues [20,34], affecting the level of hormonal regulation and the amount of endorphins, cortisol, and melatonin in the human body. With an appropriate increase in endorphin and melatonin content and a decrease in cortisol content, the human body is in a state of relaxation and pleasure, and the strength of the immune response system is strengthened, so it is possible to sleep smoothly and eliminate the symptoms of insomnia [35,36,37,38]. On the other hand, the ingredients of agarwood aromatherapy act on the *MAOA*, *MAOB*, and *SLC6A* family protein receptors to balance the homeostasis of 5-HT in the central nervous system by inducing 5-HT signaling in the intercellular network to maintain a long and deep sleep. It also participates in the metabolism of neuroactive and vasoactive amines in the central nervous system and peripheral tissues [39] in the catalytic degradation of dopamine and other neurotransmitters, reducing the body’s excitability and putting it in a “sleepy” state [40]. The CUCMs are additionally involved in the regulation of adrenergic receptor activity, protein kinase activator activity, and catechol hormone activity [41], which further enhances the effect on the hormonal channels for the treatment of sleep disorders. The CUCNs are more deeply involved in the metabolism of dopamine, intervening in the catabolic process of neurotransmitters, and at the same time enhancing the influence on the GABA receptor complex, 5-HT receptor complex, and neurotransmitter receptor activity, which further broadens the path of neurotransmitters in the treatment of sleep disorders [42,43].

### 2.5. Construction of the Agarwood Aromatherapy’s ‘Active Components–Efficacy Targets–Action Pathways’ Network

**Figure 9:**
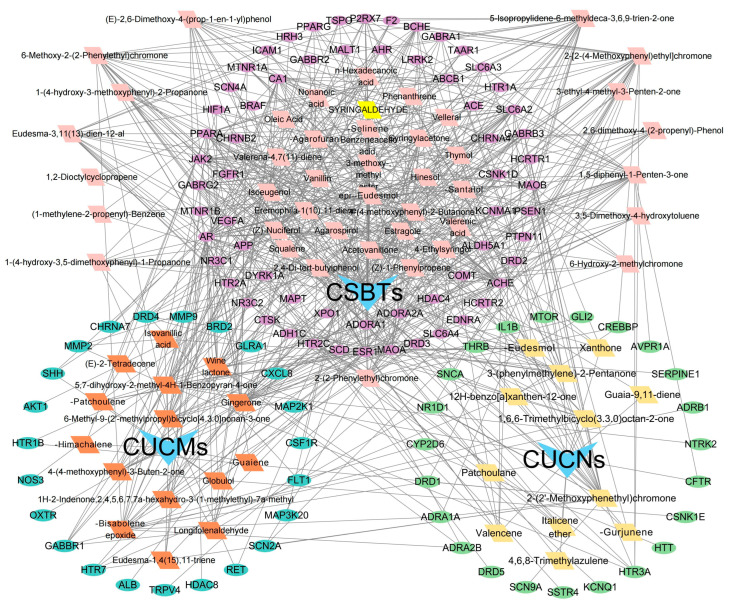
Multi-level network diagram of ‘Active Components–Efficacy Targets–Action Pathways’ in agarwood aromatherapy.

Based on the active ingredients and efficacy targets of agarwood aromatherapy, combined with the gene targets of sleep disorders, a network relationship diagram of ‘Active Components–Efficacy Targets–Action Pathways’ of agarwood aromatherapy was constructed with the help of Cytoscape 3.9.1 software. In the diagram, square nodes represent the active ingredients, round nodes represent the gene targets, and triangles of different colors represent the grouping of different active ingredients. The connecting line between an ingredient and a target indicates that the target can be regulated by this ingredient. The construction of the multi-level network of ‘Active Components–Efficacy Targets–Action Pathways’ more intuitively shows the regulation relationship between the active ingredients of agarwood aromatherapy and the targets of sleep disorder-related diseases and demonstrates the mechanism of multi-component, multi-pathway, and multi-level action (Figure 9).

### 2.6. The Analysis of the Molecular Docking

**Figure 10:**
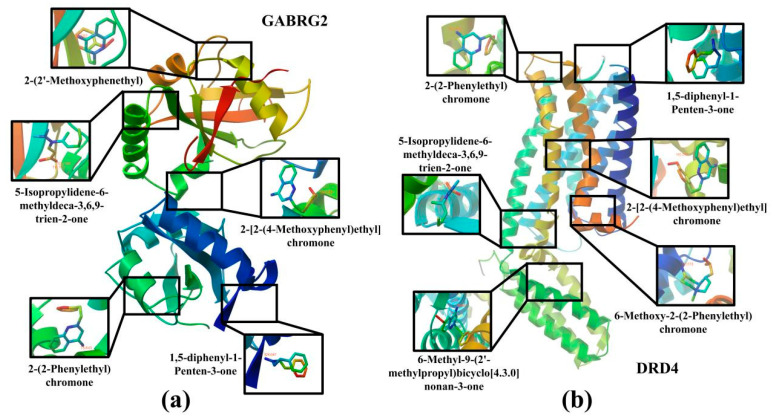
Docking diagram of the main active ingredients and core target molecules: (**a**) Molecular docking results for GABRG2 and five compounds; (**b**) molecular docking results for DRD4 and six compounds.

In order to verify the reliability of the results of the network pharmacological analysis, the degree value was used to screen out the top 5 ingredients among the CSBTs and the top 3 ingredients among the CUCMs and CUCNs, for a total of 11 active ingredients to be used as the core ingredients, which were molecularly docked with the core target of the action, respectively. The results show that 5-Isopropylidene-6-methyldeca-3,6,9-trien-2-one with *MAOB*, *GABRG2* and *DRD4*; 1,5-diphenyl-1-Penten-3-one with DRD4 and GABRG2; 2-(2-Phenylethyl)chromone with *DRD4* and *GABRG2*; and 6-Methoxy-2-(2-Phenylethyl)chromone with *DRD4* and *MAOB*, a total 21 groups of molecules with docking binding energies less than −1.2 kcal·mol^−1^. This indicates that the screened components all produced better binding to the targets, suggesting that the main active components of agarwood aromatherapy can improve sleep through multiple targets. The receptor–ligand binding patterns are shown in Table 2 (Figure 10).

## 3. Materials and Methods

### 3.1. Materials

#### 3.1.1. Experimental Instruments

5977B/8860 Agilent GC-MS (Agilent Technologies Ltd., Santa Clara, CA, USA), HWS-26 Electrothermal Thermal Bath (Shanghai Yiheng Experimental Instrument Co., Shahai, China), XS105DU Electronic Balance (Mettler Toledo Instruments Ltd., Zurich, Switzerland), SPME Fiber Assembly with PDS (No. 57300-U, Sigma-Aldrich Ltd., Tokyo, Japan), SPME Fibre Optic Holder (No. 57330-U, Sigma-Aldrich Ltd., Darmstadt, German).

#### 3.1.2. Experimental Materials

The agarwood aromatherapies used in this experiment were purchased from Guangdong Chi-Nan Mountain agarwood Co. (Maoming, China) and Yufeng Qunxianghui agarwood Co. (Maoming, China). Among them, 3 batches are ordinary agarwood aromatherapy made from ordinary agarwood, and 3 batches are Chi-Nan agarwood aromatherapy made from Chi-Nan agarwood, with specifications of (20 cm × 1.5 mm).

### 3.2. Activation of Adsorption-Extraction Units

Prior to the adsorption of agarwood aromatherapy components, the SPME Fibre Optic Holder with the PDS SPME Fiber Assembly inserts the sample inlet of 5977B/8860 Agilent GC/MS, pushes the quartz fibre with the adsorption head into the inlet, and activates the extraction head for 30 min at 50 °C in the column chamber and 240 °C in the injection port.

### 3.3. Detection of Volatile Components in Agarwood Aromatherapy

Light the agarwood aromatherapy used in the experiment and place it in a closed glass container, and insert the activated adsorption device into the container through a glass tube. Launch the quartz fiber to place it in the environment of the agarwood aromatherapy parcel, through the conduit to the glass instrument at an even pace so that the agarwood aromatherapy will not be extinguished. After 20 min of adsorption, remove the device and insert it again into the inlet port of the 5977B/8860 Agilent GC-MS instrument, connected to the instrument, to run the program and conduct the analysis of component determination.

### 3.4. GC-MS Analysis 

#### 3.4.1. Chromatographic Conditions

The separation of compounds was achieved on HP-5MS Flexible Quartz Capillary Columns (30 mm × 0.25 mm, 0.25 μm). This analysis process used high-purity helium as a carrier gas with the volume flow in 1 mL/min, the inlet temperature was 250 °C, and the detector temperature was 300 °C. The temperature programming was as follows: the start temperature was 50 °C for 1 min, rising to 143 °C at the speed of 15 °C/min and then holding for 10 min. Then, the temperature rose at 1 °C/min until it reached 155 °C, followed by rising to 225 °C at the speed of 25 °C/min and holding for 7 min. Finally, the temperature was increased to 250 °C at a rate of 25 °C/min and maintained for 10 min. The mass spectrometer was run in 70 eV electron, the ion source temperature was 250 °C, and the scanning range was from 50 to 500 *m*/*z*.

#### 3.4.2. Analysis of Sesquiterpene Components

After GC-MS analysis, the mass spectral data of agarwood EO components were searched using NIST SEARCHER 2.4 in combination with the NIST17 database, and the relative contents of each component were calculated according to the peak area normalization method.

### 3.5. Network Pharmacology and Analysis

#### 3.5.1. Confirmation of Active Ingredients and Prediction of Targets in Agarwood Aromatherapy

Based on the information of the aroma components of the different types of agarwood aromatherapy obtained from GC-MS analysis, they were classified into three parts, components shared by both types of agarwood (CSBTs), components unique to ordinary agarwood (CUCMs), and components unique to Chi-Nan agarwood (CUCNs). We used the PubChem database to query Smiles numbers for individual chemical components, then we searched the relevant target databases Swiss Target Prediction [44] (https://www.swisstargetprediction.ch/, accessed on 4 January 2024), ChEMBL [45] (https://www.ebi.ac.uk/chembl/, accessed on 4 January 2024), and TargetNet [46] (https://www.targetnet.scbdd.com/, accessed on 4 January 2024) to build the potential targets of the agarwood aromatherapy components. Finally, the gene names of the target data were normalized using UniProt [47] (http://www.uniprot.org/, accessed on 4 January 2024) and only the human origin (Homosapiens) target data were retained for subsequent analysis.

#### 3.5.2. Construction of the Collection of Targets Related to Sleep Disorders

The components of burnt agarwood aromatherapy have excellent sleep-aiding effects, so this study further searched the genes of sleep disorders based on GeneCards [48] (https://www.genecards.org/, accessed on 5 January 2024), Open Target Platform [49] (www.targetvalidation.org/, accessed on 5 January 2024), and DisGeNet [50] (https://www.disgenet.org/, accessed on 5 January 2024). ‘Insomnia’ and ‘Sleep disorders’ were used as keywords to search for disease targets related to sleep disorders, and the names of the genes were standardized by Integrated Discovery (DAVID) v6.8 [51] (https://david.abcc.ncifcrf.gov/, accessed on 5 January 2024). To ensure the credibility of the data, only correlation values ≥ 1.5 were selected as core target information for sleep disorders.

#### 3.5.3. Construction and Analysis of Protein–Protein Interaction Network

The JVenn [52] (http://jvenn.toulouse.inra.fr/app/example.html/, accessed on 5 January 2024) platform was used to screen the target genes of the aroma components of each part of the agarwood aromatherapy with the disease targets related to sleep disorders, removing the duplicates and selecting the intersections as the potential targets of the agarwood aromatherapy to improve sleep. The obtained collection of target genes was submitted to STRING [53] (https://cn.string-db.org/, accessed on 5 January 2024) for the construction of a PPI network. Using Homo sapiens as the target species, and selecting a high confidence level of 0.7 as the threshold for the interactions score, the PPI network obtained under this condition was visualized in Cytoscapev 3.9.1, and the top 10 core targets in each section were screened according to the Degree value using the CytoNCA plug-in. 

#### 3.5.4. Construction of the ‘Active Components–Efficacy Targets–Action Pathways’ Network

In Cytoscape 3.9.1, a multilevel network of ‘Active Components–Efficacy Targets–Action Pathways’ was constructed to associate the active components, efficacy targets, and disease genes of three parts of the agarwood aromatherapy. The circle represents the target of efficacy, the diamond represents the active ingredient, and the triangle represents the group of ingredients to establish the network and analyze the effects of the agarwood on improving sleep.

#### 3.5.5. GO Functional Analysis and KEGG Pathway Enrichment Analysis

The potential targets of the three parts of the agarwood aromatherapy components corresponding to the improvement of sleep were imported into the Metascape [54] (https://metascape.org/, accessed on 6 January 2024) and David databases [55] (https://david.ncifcrf.gov/, accessed on 6 January 2024). The databases conducted Gene Ontology (GO) and Kyoto Encyclopedia of Genes and Genomes (KEGG) pathway enrichment analyses to obtain biological information about potential targets and analyze the potential mechanism of action of agarwood aromatherapy in improving sleep. GO analysis results are divided into the categories of biological processes (BPs), cellular compounds (CCs), and molecular functions (MFs). According to the *p*-values, the top 10 results from GO analysis were selected and a histogram of enrichment quantity statistics was drawn; for KEGG analysis, the top 10 pathways were selected for visualization [11,56].

#### 3.5.6. Molecular Docking Validation

Molecular docking was carried out for each part of the agarwood aromatherapy active ingredients and screened core targets to predict and obtain the binding energy of protein–molecule interactions to determine whether they are well bound or not. The 3D structures of the small molecules were obtained using TCMSP [56] (https://www.tcmsp-e.com/tcmsp.php/, accessed on 7 January 2024) and PubChem [57] (https://pubchem.ncbi.nlm.nih.gov/, accessed on 7 January 2024) databases, and the PDB files of the 3D structures of the core targets were obtained using Open Bable GUI for format conversion and RCSB database [58] (https://www.rcsb.org/, accessed on 7 January 2024). Molecular docking was carried out for each part of the agarwood aromatherapy active ingredients and screened core targets to predict and obtain the binding energy of protein–molecule interactions to determine whether they are well bound or not. The 3D structures of the small molecules were obtained using TCMSP and PubChem databases, and the PDB files of the 3D structures of the core targets were obtained using Open Bable GUI for format conversion and RCSB database (https://www.rcsb.org/, accessed on 7 January 2024). They were dehydrogenated using PyMol software (PyMol 2.5.0), then docked by AutodockTool software (AutodockTool 1.5.7), and finally the structures were visualized again using PyMol. The binding energy was used as an indicator to evaluate the binding activity and docking effects of ligand–protein interactions, and it is generally accepted that a binding energy less than −1.2 KCal·mol^−1^ indicates a strong binding of the two [59].

## 4. Conclusions

### 4.1. Differences in the Chemical Composition of Agarwood Aromatherapy between Ordinary and Chi-Nan Agarwood

Agarwood aromatherapy primarily consists of sesquiterpenes, small aromatic compounds, chromones, and a small amount of fatty acid compounds. While the composition of aromatherapy within each group of ordinary agarwood and Chi-Nan agarwood is consistent, there are significant differences between these two types of agarwood. In ordinary agarwood aromatherapy, sesquiterpenes and small aromatic substances constitute about 60% of the total composition, whereas Chi-Nan agarwood aromatherapy is predominantly composed of chromones.

Among the 18 unique components of common agarwood (CUCMs), the sesquiterpene α-Curcumene is the most abundant. α-Curcumene has been shown to significantly inhibit the migration and invasion of MDA-MB-231 breast cancer cells by suppressing matrix metalloproteinase-9 (MMP-9), demonstrating excellent therapeutic potential in the treatment of breast cancer [60,61]. Other notable sesquiterpenes in ordinary agarwood include β-Patchoulene, α-Himachalene, α-Bisabolene epoxide, and α-Guaiene. β-Patchoulene, for example, has been found to protect the intestinal mucosal barrier by reducing apoptosis, decreasing tissue and serum levels of lipopolysaccharide-binding protein (LBP), and modulating key signaling pathways such as ROCK-MLC and TLR4-mediated NF-κB, thereby regulating immune function and mitigating intestinal mucosal damage [62]. α-Bisabolene epoxide is another significant component, known for its antilipogenic activity and selective antibacterial properties against Gram-positive bacteria [63]. Additionally, isovanillic acid, a fatty acid compound unique to ordinary agarwood, has anticoagulant properties, promoting the inhibition of pro-coagulant protease and fibrinolytic enzyme activities, thereby prolonging clotting time [64].

In contrast, Chi-Nan agarwood aromatherapy features 18 unique components (CUCNs), with 5-[(4-Hydroxy-3-methoxyphenyl)methyl]-1,3-dimethyl-1,3-diazinane-2,4,6-trione and Italicene ether being the most prominent, together accounting for approximately 6% of the composition. Notably, Kessane and β-Elemene are exclusive to Chi-Nan agarwood. Kessane is known for its ability to inhibit central nervous system activity, reduce nervous excitability, and improve sleep quality [5,65]. β-Elemene, which has a distinctive aniseed odor, is effective in directly killing tumor cells without affecting normal cells at conventional dosages [66]. It induces apoptosis by disrupting the growth and metabolism of tumor cells, particularly by blocking the cell cycle at the S-phase, preventing cells from progressing to the G2 and M phases, thus inhibiting cell proliferation [67]. Furthermore, Chi-Nan agarwood is rich in chromones, with 2-(2-Phenylethyl)chromone and 2-[2-(4-Methoxyphenyl)ethyl]chromone being the most prevalent. These chromones have demonstrated significant inhibitory effects on tumor cells, making them potent cancer inhibitors [68,69].

Overall, the distinct chemical compositions of ordinary and Chi-Nan agarwood aromatherapies reflect their different therapeutic properties, with ordinary agarwood focusing more on the regulation of neurotransmitters and immune function, while Chi-Nan agarwood emphasizes anti-cancer properties and central nervous system regulation.

### 4.2. Differences and Similarities in the Mechanisms of Sleep-Improving Effects of Ordinary and Chi-Nan Agarwood Aromatherapies

In the case of Chi-Nan agarwood and ordinary agarwood aromatherapies, both have the effect of improving sleep, the CSBTs that improve sleep from both hormonal and neurotransmitter pathways. On the one hand, by regulating the content of endorphins, cortisol, melatonin, and other hormone components in the human body so that the human body is in a relaxed and happy atmosphere, reducing the pressure on the nerves and ‘induced’ into dreaming. On the other hand, the ordinary ingredients act through the regulation of neurotransmitters in the body’s activities so that 5-HT is in a homeostatic state, and the catalytic degradation of dopamine reduces the body’s excitability for the human body to quickly enter the sleep state. The CUCMs can additionally participate in the action pathway of adrenaline activity and catechol hormone activity; adrenaline, as a hormone and neurotransmitter, will make the heart contraction force rise so that the heart, liver, and tendon vasodilatation of blood vessels will lead to faster heartbeat and higher blood pressure and thus cause insomnia symptoms [70]. Gingerone, 5,7-dihydroxy-2-methyl-4H-1-Benzopyran-4-one,6-Methyl-9-(2′-methylpropyl)bicyclo [4.3.0]nonan-3-one and other key ingredients reduce the secretion of adrenaline by acting on the target genes *COMT*, *DRD4*, and *ESR1*, lowering the level of exciting hormones in the blood circulation and improving the sleep condition from the source [20,71,72]. While the Chi-Nan agarwood aromatherapy is more involved in the metabolic process of neurotransmitters, 2-(2′-Methoxyphenethyl)chromone, γ-Eudesmol, Valencene, and other active ingredients, through the action of the core target *HTR1B*, activate the protein receptors of 5-HT, which in turn transmits signals to the SERT synapse to activate the *SERT*, accelerating the process of serotonin transport, awakening the *MAOA* target to regulate the production and metabolism of 5-HIAA [73,74], and also acting on another core target, the *GABRG2*-associated receptor protein, as a “switch” for transport and ion passage, playing a key role in the formation of functionally inhibitory GABAergic synapses [24].

In conclusion, the present study compares the differences in chemical composition between ordinary agarwood and Chi-Nan agarwood aromatherapies, then explores the mechanism of action of the two types of agarwood aromatherapy to improve sleep through network pharmacology and molecular docking techniques. The experimental results show that the chemical compositions between different agarwood aromatherapies are different; both types of agarwood contain a large number of small molecules of aromatic substances and sesquiterpenes, but the Chi-Nan agarwood aromatherapy contains a higher content of chromone components, and the different types and levels of ingredients are also the main reason why the two types of agarwood aromatherapy focus on different mechanisms of action to improve sleep. At the same time, through more detailed web-based pharmacological analyses, this study also found that agarwood aromatherapy contains many antidepressant aspects while improving sleep, which provides some reference value for subsequent research on antidepressant aspects of agarwood aromatherapy.

## Figures and Tables

**Table 1:** Chemical compounds of agarwood aromatherapy.

No.	Compounds	Chemical Formula	MW	Relative Percentage of Agarwood Aromatherapy Compounds (%)					
				CX01	CX02	CX03	QN01	QN02	QN03
1	α-Agarofuran	C_15_H_24_O	220.35	1.24	1.53	1.37	2.57	2.52	1.88
2	α-Santalol	C_15_H_24_O	220.35	1.27	1.13	1.27	1.83	0.81	1.11
3	epi-γ-Eudesmol	C_15_H_26_O	222.37	1.18	0.96	0.81	0.41	0.83	0.63
4	Agarospirol	C_15_H_26_O	222.37	0.34	0.65	0.26	0.46	0.23	0.30
5	Hinesol	C_15_H_26_O	222.37	0.12	0.19	0.17	0.39	0.28	0.28
6	β-Gurjunene	C_15_H_24_	204.35	1.53	2.19	1.07	1.05	0.58	0.65
7	Eudesma-3,11(13)-dien-12-al	C_15_H_22_O	218.33	0.96	-	-	0.57	-	0.30
8	α-Selinene	C_15_H_24_	204.35	0.46	0.54	0.44	0.31	0.44	0.56
9	Longifolene	C_15_H_24_	204.35	0.90	1.11	0.94	0.65	0.37	0.54
10	Aromandendrene	C_15_H_24_	204.35	0.40	0.46	0.64	0.28	0.37	0.60
11	Alloaromadendrene	C_15_H_24_	204.35	1.27	0.96	0.47	0.98	0.49	0.75
12	Valerenic acid	C_15_H_22_O_2_	234.33	0.68	0.69	0.68	0.41	0.49	0.81
13	Velleral	C_15_H_20_O_2_	232.32	1.61	0.19	-	0.78	-	0.42
14	(Z)-Nuciferol	C_15_H_22_O_2_	260.39	0.40	1.30	-	0.39	0.56	0.51
15	Eremophila-1(10),11-diene	C_15_H_24_	204.35	0.86	1.21	1.12	0.79	0.76	1.44
16	Valerena-4,7(11)-diene	C_15_H_24_	204.35	0.65	1.07	1.46	0.17	0.19	0.11
17	(4ar-cis)-2(3H)-Naphthalenone 4,4a,5,6,7,8-hexahydro-4a,5-dimethyl-3-(1-methylethylidene)	C_15_H_22_O	218.33	-	0.38	0.51	0.54	0.62	0.84
18	Hydroquinone	C_6_H_6_O_2_	110.11	1.18	0.69	1.11	0.65	-	0.74
19	(1-methylene-2-propenyl)-Benzene	C_10_H_10_	130.19	0.56	0.84	0.72	1.38	1.39	0.54
20	Vanillin	C_8_H_8_O_3_	152.15	3.84	-	4.10	2.64	2.85	2.21
21	(Z)-1-Phenylpropene	C_9_H_10_	118.18	-	0.84	-	-	-	0.25
22	Estragole	C_10_H_12_O	148.20	-	0.42	-	-	-	0.93
23	3,5-Dimethoxy-4-hydroxytoluene	C_9_H_12_O_3_	168.19	5.14	9.55	9.79	1.22	2.04	1.67
24	Isoeugenol	C_10_H_12_O_2_	164.20	13.99	10.12	15.15	1.01	1.71	1.23
25	Acetovanillone	C_9_H_10_O_3_	166.17	4.80	7.44	5.22	0.78	0.72	1.81
26	4-(4-methoxyphenyl)-2-Butanone	C_11_H_14_O_2_	178.23	2.35	2.42	2.65	-	-	0.46
27	2,4-Di-tert-butylphenol	C_14_H_22_O	206.32	1.65	-	2.53	1.68	1.09	1.39
28	4-Ethylsyringol	C_10_H_14_O_3_	182.22	3.31	2.91	3.93	-	4.88	4.69
29	1-(4-hydroxy-3-methoxyphenyl)-2-Propanone	C_10_H_12_O_3_	180.20	1.42	1.92	1.62	0.42	-	0.42
30	Nonanoic acid	C_9_H_18_O_2_	158.24	1.23	0.96	-	0.35	-	-
31	Vinylsyringo	C_10_H_12_O_3_	180.20	15.86	13.04	16.55	1.94	1.85	2.16
32	5-Isopropylidene-6-methyldeca-3,6,9-trien-2-one	C_14_H_20_O	204.31	0.27	-	0.49	0.54	0.60	-
33	Benzeneacetic acid, 3-methoxy-, methyl ester	C_10_H_12_O_3_	180.20	0.10	-	-	0.50	0.51	0.39
34	2,6-dimethoxy-4-(2-propenyl)-Phenol	C_11_H_14_O_3_	194.23	2.77	2.88	2.06	0.41	0.79	0.81
35	(E)-2,6-Dimethoxy-4-(prop-1-en-1-yl)phenol	C_11_H_14_O_3_	194.23	-	5.18	1.97	1.18	-	0.91
36	SYRINGALDEHYDE	C_7_H_6_O_2_	182.17	3.72	4.87	2.99	0.61	-	0.98
37	Thymol	C_10_H_14_O	150.22	0.47	0.33	0.36	0.74	0.74	0.65
38	Phenanthrene	C_14_H_10_	178.23	-	0.11	0.13	0.44	-	-
39	Syringylacetone	C_11_H_14_O_4_	224.25	1.27	2.22	1.17	0.37	0.21	0.63
40	3-ethyl-4-methyl-3-Penten-2-one	C_8_H_14_O	126.20	0.40	-	-	0.87	0.49	1.19
41	1-(4-hydroxy-3,5-dimethoxyphenyl)-1-Propanone	C_11_H_14_O_4_	210.23	0.65	0.77	0.34	-	0.28	-
42	6-Hydroxy-2-methylchromone	C_10_H_8_O_3_	176.17	0.11	-	0.21	0.52	0.44	0.37
43	1,2-Dioctylcyclopropene	C_19_H_36_	264.50	0.25	0.46	-	-	0.51	-
44	n-Hexadecanoic acid	C_16_H_32_O_3_	256.42	1.58	0.84	1.41	1.07	2.20	1.26
45	Oleic Acid	C_18_H_34_O_2_	282.50	0.15	-	-	0.11	0.21	0.16
46	1,5-diphenyl-1-Penten-3-one	C_17_H_16_O	236.31	0.12	0.15	-	0.39	0.39	0.54
47	2-(2-Phenylethyl)chromone	C_17_H_14_O_2_	250.29	2.54	2.86	2.82	31.65	32.04	32.20
48	6-Methoxy-2-(2-Phenylethyl)chromone	C_18_H_16_O_3_	280.30	1.33	0.84	0.65	0.72	0.67	0.21
49	2-[2-(4-Methoxyphenyl)ethyl]chromone	C_18_H_16_O_3_	280.30	1.20	1.34	1.58	22.83	20.56	17.78
50	2-hydroxy-1,2-bis(4-methoxyphenyl)-Ethanone	C_16_H_16_O_4_	272.29	0.87	-	0.41	0.20	0.49	0.19
51	Squalene	C_30_H_50_	410.70	0.68	0.46	0.77	0.33	1.23	0.19
52	β-Patchoulene	C_15_H_24_	204.35	0.22	0.35	0.41	-	-	-
53	α-Himachalene	C_15_H_24_	204.35	0.55	0.63	0.45	-	-	-
54	α-Curcumene	C_15_H_22_	202.33	4.33	5.02	2.01	-	-	-
55	α-Bisabolene epoxide	C_15_H_24_	220.35	0.43	0.34	0.22	-	-	-
56	α-Guaiene	C_15_H_24_	204.35	0.31	0.81	0.73	-	-	-
57	Longifolenaldehyde	C_15_H_22_O	220.35	0.20	-	-	-	-	-
58	Globulol	C_15_H_26_O	222.37	0.56	0.31	0.62	-	-	-
59	Eudesma-1,4(15),11-triene	C_15_H_22_	202.33	0.32	-	-	-	-	-
60	Isovanillic acid	C_8_H_8_O_4_	168.15	0.46	-	-	-	-	-
61	9-hydroxy-Eremophila-7(11),9-dien-8-one	C_15_H_22_O_2_	238.35	0.06	-	0.12	-	-	-
62	Gingerone	C_11_H_14_O_3_	194.23	0.42	-	0.11	-	-	-
63	1H-2-Indenone,2,4,5,6,7,7a-hexahydro-3-(1-methylethyl)-7a-methyl	C_13_H_20_O	192.30	0.62	0.58	0.31	-	-	-
64	Di(2-furyl)ketone	C_9_H_6_O_3_	162.14	-	0.26	0.22	-	-	-
65	5,7-dihydroxy-2-methyl-4H-1-Benzopyran-4-one	C_10_H_12_O_3_	192.17	0.31	-	0.13	-	-	-
66	Wine lactone	C_10_H_14_O_2_	166.22	-	0.30	-	-	-	-
67	(E)-2-Tetradecene	C_14_H_28_	196.37	1.97	1.50	1.80	-	-	-
68	4-(4-methoxyphenyl)-3-Buten-2-one	C_11_H_12_O_2_	176.21	0.84	0.11	0.23	-	-	-
69	6-Methyl-9-(2′-methylpropyl)bicyclo [4.3.0]nonan-3-one	C_14_H_24_O	208.34	1.08	0.76	0.69	-	-	-
70	5-[(4-Hydroxy-3-methoxyphenyl)methyl]-1,3-dimethyl-1,3-diazinane-2,4,6-trione	C_14_H_14_N_4_O_5_	292.29	-	-	-	3.28	2.38	3.18
71	12H-benzo[a]xanthen-12-one	C_17_H_10_O_2_	246.26	-	-	-	0.15	0.05	0.09
72	2-(2′-Methoxyphenethyl) chromone	C_18_H_16_O_3_	280.30	-	-	-	-	0.71	0.86
73	Xanthone	C_13_H_8_O_2_	196.20	-	-	-	0.66	0.51	0.53
74	1,6,6-Trimethylbicyclo(3.3.0)octan-2-one	C_11_H_18_O	166.26	-	-	-	0.18	0.11	-
75	3-(phenylmethylene)-2-Pentanone	C_12_H_14_O	174.24	-	-	-	-	0.23	0.39
76	4,6,8-Trimethylazulene	C_13_H_14_	170.25	-	-	-	-	0.79	-
77	[1S-(1R*,9S*)]-10,10-dimethyl-2,6-bis(methylene)-Bicyclo [7.2.0]undecane	C_15_H_24_	204.35	-	-	-	0.41	0.51	0.77
78	Guaia-9,11-diene	C_15_H_24_	204.35	-	-	-	0.72	0.69	0.72
79	Italicene ether	C_15_H_24_O	220.35	-	-	-	2.33	3.01	1.98
80	Eremophila-9(10),11(12)-diene	C_15_H_24_	204.35	-	-	-	1.05	0.51	0.72
81	Eudesma-4(14),(11)-diene	C_15_H_24_	204.35	-	-	-	-	-	0.28
82	Patchoulane	C_15_H_26_	206.37	-	-	-	-	-	0.30
83	γ-Gurjunene	C_15_H_24_	204.35	-	-	-	0.87	0.42	-
84	γ-Eudesmol	C_15_H_26_O	222.37	-	-	-	0.61	0.42	0.53
85	β-Elemene	C_15_H_24_	204.35	-	-	-	0.46	-	0.33
86	Valencene	C_15_H_24_	204.35	-	-	-	0.54	0.42	-
87	Kessane	C_15_H_26_O	222.37	-	-	-	0.66	0.86	1.04

**Table 2:** Information related to the molecular docking of the main active ingredient and core targets.

Main Active Ingredients	Core Targets	Binding Energy kcal·mol^−1^	Main Active Ingredients	Core Targets	Binding Energy kcal·mol^−1^
5-Isopropylidene-6-methyldeca-3,6,9-trien-2-one	MAOB	−5.32	2-[2-(4-Methoxyphenyl)ethyl] chromone	DRD4	−5.10
	GABRG2	−4.80		CREBBP	−6.94
	DRD4	−4.02	Gingerone	ESR1	−5.88
1,5-diphenyl-1- Penten-3-one	GABRG2	−5.74		MAOB	−4.37
	DRD4	−6.59		COMT	−4.53
2-(2-Phenylethyl) chromone	DRD4	−5.94	5,7-dihydroxy-2-methyl-4H-1-Benzopyran-4-one	ESR1	−4.98
	GABRG2	−5.73	6-Methyl-9-(2′-methylpropyl)bicyclo [4.3.0]nonan-3-one	DRD4	−5.97
6-Methoxy-2-(2-Phenylethyl) chromone	MAOB	−5.42	2-(2′-Methoxyphenethyl) chromone	HTR1B	−5.32
	DRD4	−4.93		GABRG2	−5.24
2-[2-(4-Methoxyphenyl)ethyl] chromone	HIF1A	−7.08	γ-Eudesmol	ESR1	−6.76
	GABRG2	−6.31			

## Data Availability

All the data have appeared in the paper. Data will be available upon request to the corresponding authors.
